# Keyword Index

**DOI:** 10.1038/sj.bjc.6600444

**Published:** 2002-06-17

## Abstract

*British Journal of Cancer* (2002) **86**, 1981–1985. DOI: 10.1038/sj.bjc.6600444 www.bjcancer.com

© 2002 Cancer Research UK

